# Cutaneous lesions as the first manifestation of breast cancer: a rare case

**DOI:** 10.11604/pamj.2020.37.383.27380

**Published:** 2020-12-29

**Authors:** Harwin Prestasia Putra, Khairuddin Djawad, Airin Riskianty Nurdin

**Affiliations:** 1 Department of Dermatology and Venereology, Hasanuddin University, Dr. Wahidin Sudirohusodo Central Public Hospital, Makassar, Indonesia

**Keywords:** Breast cancer, case report, cutaneous metastasis, first manifestation

## Abstract

Cutaneous metastases due to internal malignancies are rare but, in some cases, may present as the first manifestation of an undiagnosed malignancy. A 62-year-old female presented with itchy reddish patch on the left neck which spread to the left shoulder, arm, breast, and back four weeks prior to admission. Ten days later, vesicles emerged, ruptured, and ulcerated. The patient denied any history of breast lump. Skin biopsy showed diffuse dermal nests of tumor cells infiltrating to the collagen bundles and fat tissue, which was consistent with cutaneous metastasis from the breast cancer. The patient refused further workup and treatment and eventually passed away. This case showed the importance of conducting a comprehensive evaluation in cutaneous lesions presenting with early or sudden onset, rapid evolution, tendency to bleed, that do not resolve with treatment.

## Introduction

Cutaneous metastasis (CM) is defined as a neoplastic lesion that arise from a primary malignancy affecting the dermis or subcutaneous tissue. Basic patterns of cutaneous and subcutaneous tissue infiltration can result from: mechanical tumor stasis (anatomical proximity and lymphatic drainage), organ-specificity (selective affinity of tumor cells to a specific organ) or can be nonselective (independent of mechanical and organ-specific factors) [1, 2]. The frequency of the different histotypes of metastases are usually related to the primary malignancy according to age and gender. In women, cutaneous metastases most often originate from breast, ovary, oral cavity, lung, and large intestine primary malignancies. The head, neck, and trunk are commonly involved sites but rarely the limbs [1]. In most cases, CM develops after the diagnosis of the primary internal malignancy and late in the course of the disease. Internal malignancies causing cutaneous metastases are very rare and have been estimated to develop in only about 0.6-10.4%, and in some cases representing the first manifestation of an internal and misdiagnosed malignancy, which is considered a rare dermatological event. With the increasing incidence of internal cancer, dermatologists may be the first to discover the disease. A high clinical suspicion is important for identifying cutaneous metastatic lesions [1, 2].

Cutaneous metastasis may present with different clinical features; typical features are painless nodules with firm or elastic consistency which develop within a short time [1, 3]. In other cases, they may mimic specific inflammatory or neoplastic dermatological conditions which frequently cause a diagnostic delay and even misdiagnosis [1, 4]. Diagnosis of CM is usually suspected before performing a biopsy. However, this can be a pitfall for clinicians when the clinical presentation is not the typical inflammatory nodule or mass. Identifying these manifestations in patients with no previous diagnosis of a primary neoplasia, as well as the appropriate management of these uncommon lesions, is a real challenge for the dermatologist [5, 6]. Cutaneous metastases are associated with poor prognosis, higher staging of primary malignancies, recurrence, and, if they arise during therapy, indicate an inadequate response [7]. Because skin metastases are more easily recognized and detected earlier than metastasis in other organs, dermatologists and pathologists must be vigilant and be able to recognize this.

## Patient and observation

**Figure 1:**
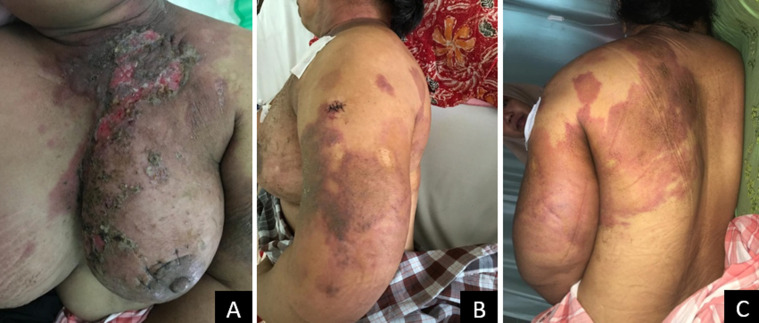
cutaneous manifestation of breast cancer; A) ulcerated lesions with erythematous patches and crusts on the left breast; B and C) erythematous macules on the left back and arm

**Figure 2:**
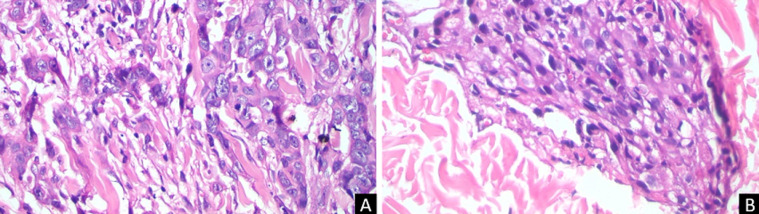
histopathology from 2 different sites with same features, carcinoma cell nests with very prominent and pleomorphic nucleoli, more than 20 mitoses per 10 high power fields, carcinoma cell nests infiltrate to connective and fat tissue; A) tissue from breast site, B) tissue from left arm site

A 62-year-old female was referred to our dermatology department for evaluation of itchy reddish patch that had appeared on the left neck for four weeks which spread to the left shoulder, left arm, chest, left breast, and left back. Ten days later, vesicles emerged and ruptured, leading to the formation of ulcer. The ulcer on the left breast became purulent and was accompanied by severe pain, swelling, and unpleasant odor. The patient denied any history of previous lump in the breast. There was no history of breast tumors in the patient's family. The patient was suspected of having deep mycosis or cutaneous T-cell lymphoma. Based on the patient medical history and clinical features, we performed skin biopsies on two different sites: ulcer on the breast and reddish patch on the left arm (Figure 1, Figure 2).

Physical examination showed erythematous scaly plaques on the left thoracic region, left arm, and left portion of the posterior trunk. In addition, a foul-smelling purulent ulcer was observed on the left breast. Histopathology examinations of both sites showed diffuse dermal nests of tumor cells infiltrating to the collagen bundles and fat tissue. Tumor cells were round with little pleomorphic nuclei and very prominent nucleoli. The mitotic count was more than 20 mitoses per 10 high power fields with 20% of the tumor cells forming tubular appearance. Based on the clinical findings and histopathological examination results, a final diagnosis of cutaneous metastasis of breast cancer was made and the patient was referred to the department of Oncology for further evaluation and treatment. Unfortunately, the patient was not willing to take chemotherapy and eventually passed away one month later.

## Discussion

The incidence of cutaneous metastasis has increased due to higher cancer survival rates and better treatment alternatives. Due to the large variability in clinical course, diagnosis of both the skin metastases and the primary tumor can be complicated [2]. In 2013, a Brazilian study demonstrated that the most common sites of cutaneous metastases are the chest (31.94% of cases) followed by the abdomen (20.37%) and scalp (14.81%). In women, the most common primary malignancy was breast cancer (63.19%), intestines (10.41%) and lung (4.16%), which tends to metastasize later to the anterior thoracic wall [2, 6]. Although cutaneous metastasis most frequently originates from breast cancer, it rarely occurs as the first manifestation of this malignancy [6, 8]. Our case showed a sudden development of multiple erythematous patches that rapidly ulcerated. Cutaneous metastases from breast cancer are most commonly reported as multiple, firm, hyperpigmented, painless nodules, followed by papules, erysipelas-like, alopecic, and sclerotic plaques which may retract the skin [2, 6]. In a review of 164 patients, the most frequent manifestations were papules and/or nodules (80%), followed by telangiectatic carcinoma (11.2%), erysipeloid carcinoma (3%), carcinoma en cuirasse (3%), alopecia neoplastica (2%) and zosteriform pattern (0.8%) [8]. In some cases, lesions may be followed by the development of ulcer and may be challenging to diagnose, especially when prior history of breast cancer is absent, as presented in our case.

Histopathology remains the main diagnostic tool for cutaneous metastatic lesions. In our patient, we performed skin biopsies on two different sites, from the ulcer on the breast and reddish patch on the left arm. The result on both sites showed identical findings, diffuse dermal nests of tumor cells infiltrating to the collagen bundles and fat tissue, which confirmed that cutaneous metastasis had reached to the upper arm region. Skin biopsy is the gold standard for diagnosis and should always be considered in case of early or sudden onset, delayed healing, bleeding tendency, or vascular appearance of the lesions that do not resolve after treatment [2, 6]. Several histopathologic features may help to identify the source of the primary tumor. Microscopically, the collection of neoplastic cells, which usually resemble the primary malignancy, are generally seen in the dermis and/or subcutaneous tissue [9] Adenocarcinoma is the most common tumor type. An analysis of 124 cases of metastases revealed this characteristic in 76.6% cases. The most frequent subtypes for the primary malignancies of the breast are ductal and lobular [6]. Basic patterns of metastasis mechanisms from a breast cancer to the skin are reported to occur through direct extension, hematologic dissemination, or surgical implantation [8].

The presence of cutaneous metastasis in cases of breast malignancy may indicate advanced disease and a poor prognosis. The estimated mean survival rate after the diagnosis of cutaneous metastasis is 50% at 6 months, although patients with breast malignancy may have a better prognosis than those with other cancers [6, 9]. Poor outcome was shown in this case where the patient passed away one month after the diagnosis was made. Multidisciplinary evaluation is essential in the treatment of cutaneous metastasis to determine the extension of the process and start adequate treatment. Possible treatment includes cryotherapy, radiotherapy and chemotherapy [6, 9].

## Conclusion

This case showed the importance of conducting a comprehensive evaluation in cutaneous lesions presenting with early or sudden onset, rapid evolution, tendency to bleed, that do not resolve with treatment. Early diagnosis of cutaneous metastasis is crucial to devise an optimum treatment strategy and improve life expectancy rate.
